# Regional brain gray matter volume in world-class artistic gymnasts

**DOI:** 10.1186/s12576-020-00767-w

**Published:** 2020-09-18

**Authors:** Makoto Fukuo, Koji Kamagata, Mana Kuramochi, Christina Andica, Hiroyuki Tomita, Hidefumi Waki, Hidenori Sugano, Yuichi Tange, Takumi Mitsuhashi, Wataru Uchida, Yuki Takenaka, Akifumi Hagiwara, Mutsumi Harada, Masami Goto, Masaaki Hori, Shigeki Aoki, Hisashi Naito

**Affiliations:** 1 Juntendo University Graduate School of Health and Sports Scienc, 1-1 Hirakagakuendai, Inzai, Chiba 270-1695 Japan; 2 Department of Radiology, Juntendo University Graduate School of Medicin, Tokyo, Japan; 3 Department of Radiological Sciences, Tokyo Metropolitan University, Tokyo, Japan; 4 Department of Neurosurgery, Juntendo University Graduate School of Medicine, Tokyo, Japan; 5 Department of Radiology, University of Tokyo Graduate School of Medicine, Tokyo, Japan; 6 School of Allied Health Science, Kitasato University, Kanagawa, Japan

**Keywords:** Magnetic resonance imaging, Volume-based morphometry, Brain plasticity, Difficulty score

## Abstract

The relationship between long-term intensive training and brain plasticity in gymnasts has recently been reported. However, the relationship between abilities in different gymnastic events and brain structural changes has not been explored. This study aimed to evaluate the correlation between world-class gymnasts (WCGs)’ specific abilities in different gymnastics events and their gray matter (GM) volume. Ten right-handed Japanese male WCGs and 10 right-handed gender- and age-matched controls with no history of gymnastic training participated in this study. Whole brain three-dimensional T1-weighted images (magnetization-prepared rapid gradient-echo sequence) with 0.90 mm^3^ voxels were obtained using a 3 T-MRI scanner from each subject. Volume-based morphometry (VolBM) was used to compare GM volume differences between WCGs and controls. We then explored the correlation between specific gymnastic abilities using different gymnastic apparatuses, and GM volume. Significantly higher GM volumes (false discovery rate-corrected *p* < 0.05) in the inferior parietal lobule, middle temporal gyrus, precentral gyrus, rostral middle frontal gyrus, and superior frontal gyrus were demonstrated in WCGs, compared with controls using VolBM. Moreover, significant positive correlations were observed between brain regions and the difficulty scores for each gymnastic event, for example, rings and inferior parietal lobule and parallel bars and rostral middle frontal gyrus. These results may reflect the neural basis of an outstanding gymnastic ability resulting from brain plasticity in areas associated with spatial perception, vision, working memory, and motor control.

## Introduction

Artistic gymnastics is a major competition in the Summer Olympic Games that has attracted increasing interest worldwide (International Gymnastics Federation: FIG, https://www.gymnastics.sport/site/). Japan is one such country with an increasing interest in artistic gymnastics. Artistic gymnastics is usually divided into men's and women's gymnastics. Men perform on 6 events: floor exercise, pommel horse, rings, vault, parallel bars, and horizontal bar. Gymnastics performances in competitions are evaluated by a panel of judges using two different scores, the D-score (difficulty of the routine) and the E-score (execution of the routine). The D-score is mainly assessed according to the difficulty of the elements performed within a routine under the conditions required for each event, whereas the E-score is defined by evaluating technical errors in the routine. The total score is calculated by adding the D-score and the E-score (FIG, https://www.gymnastics.sport/site/).

Gymnasts belonging to gymnastics clubs train to increase their skills using conventional and newly developed techniques. The amount of research on artistic gymnastics has increased in recent years, mainly focused on technical aspects or coaching/teaching methods. Until now, little is known about the anatomical and physiological characteristics of gymnasts. However, it is well known that many brain areas are activated during exercise and that neuronal plasticity, defined as the ability of neurons to change in structure and function in response to environment and experience, is induced by long-term training [1–4]. Through this process, athletes can acquire exceptional abilities in perception, stimulus discrimination, decision-making, motor preparation, and execution [1]. Indeed, evidence suggests that athletes' brains are characterized by specific functional and anatomical adaptations that facilitate information processing relevant to their specific sport [5–10].

Gymnasts at the top level begin training from childhood. Such long-term intensive training with specific movements may cause specific adaptations in brain structure and function. In fact, it has been demonstrated that Chinese gymnasts exhibit higher gray matter (GM) volume in somatosensory and visuospatial areas compared with non-gymnasts using voxel-based morphometry (VBM) analysis [11]. GM includes neurons, which consist of cell bodies, dendrites, axons, and synapses, as well as glial cells like oligodendrocytes, astrocytes, and microglia. Any microstructural changes to these cells may affect GM volume and function [2, 11]. However, the relationship between abilities in different gymnastic events and structural brain changes is unknown. Better understanding the neural basis of elite gymnastics performance may inform future training strategies and, moreover, it may provide a new method of identifying talent and assessing the development process [12].

In this study, we compare GM volume differences between Japanese world-class gymnasts (WCGs) and healthy controls with no history of intensive exercise training. Furthermore, we evaluated the correlation of WCGs’ specific abilities in different gymnastics events and GM volume. Since the E-score is more susceptible to mental and physical conditions during competition than the D-score and is influenced by the referees’ subjectivity, the D-score was used as an index of the gymnasts’ specific physical abilities acquired through long-term training. We applied volume-based morphometry (VolBM) using FreeSurfer. Compared with VBM, which explores hundreds of thousands of voxel-wise GM concentrations, VolBM demonstrates a higher level of accuracy by evaluating volumes of particular regions of interest in the brain (e.g., atlas-based regions) [13].

## Materials and methods

### Subjects

Ten right-handed Japanese WCGs (10 males; mean age, 19.9±1.3 years; age range, 18–22 years; mean years of training, 13.6 ± 2.2 years; years of training range, 10–19 years) and ten right-handed gender- and age-matched healthy controls (10 males; mean age, 20.6±1.7 years; age range, 16–22 years) without any history of gymnastics training or competition participated in this study. All WCGs in this study have won medals at gymnastics world championships. Their best D-score and E-score for each gymnastics event (floor exercise, horizontal bar, rings, vault, parallel bars, and pommel horse) obtained at a recent world gymnastics competition, are listed in Table 1. The D-score evaluates the content of a routine using three criteria: difficulty, composition requirements, and connection value, whereas the E-score evaluates technical errors in the routine. The D-score was used as an objective indicator of gymnastic ability in this study. There were no participants with a history of neurological or brain injuries or psychiatric disease. The study protocol was approved by the Ethic Committee of the Juntendo University and participants provided written informed consent before scanning.

**Table 1:** Characteristics of world-class gymnasts who participated in this study

World-class gymnasts	Recent medal records	Age (years)	Years of training (years)	D-score						
				Floor exercise	Pommel horse	Rings	Vault	Parallel bars	Horizontal bar	Mean ± SD
1	DTB team challenge 2017, bronze medal	20	14	5.3	6.0	5.6	5.2	5.8	5.6	5.58 ± 0.30
2	WC 2018 Tokyo cup, silver medal	21	14	6.0	5.8	6.0	5.6	6.2	5.1	5.78 ± 0.39
	WGC 2018, bronze medal									
3	WGC 2015, gold medal	21	12	5.9	6.4	6.1	5.2	6.3	5.7	5.93 ± 0.44
	WGC 2018, bronze medal									
4	Asian Games 2018, silver medal	21	19	6.0	6.0	5.7	5.2	6.2	5.7	5.80 ± 0.35
	Universiade 2017, gold medal									
5	WGC 2015, gold medal	22	13	6.2	5.4	5.3	5.2	5.4	5.9	5.57 ± 0.39
6	DTB team challenge 2018, bronze medal	20	10	5.9	6.3	5.6	5.2	5.7	5.2	5.65 ± 0.42
7	DTB team challenge 2018, bronze medal	19	14	5.5	5.1	5.6	5.2	5.9	6.1	5.57 ± 0.39
8	Voronin cup 2016, gold medal	19	14	5.6	5.8	5.5	4.8	6.0	5.8	5.58 ± 0.42
9	IJGC 2015, gold medal	18	14	5.9	5.7	5.0	5.6	5.5	4.8	5.42 ± 0.43
10	Asian Games 2018, silver medal	18	12	5.9	6.1	5.3	5.2	6.0	5.6	5.68 ± 0.38
	All Japan 2018, gold medal									
	Mean ± SD	19.9 ± 1.4	13.6 ± 2.3	5.82 ± 0.27	5.86 ± 0.39	5.57 ± 0.33	5.24 ± 0.23	5.90 ± 0.30	5.55 ± 0.40	
			r^2^ vs Mean	0.08	0.33	0.73**	0.002	0.69**	0.03	

				E-score						
				Floor exercise	Pommel horse	Rings	Vault	Parallel bars	Horizontal bar	Mean ± SD
				8.066	8.500	8.400	7.666	8.466	7.433	8.089 ± 0.451
				8.633	8.233	8.233	9.433	8.633	7.900	8.511 ± 0.531
				8.166	8.500	8.166	8.900	8.733	8.066	8.422 ± 0.343
				8.566	8.566	8.200	8.833	8.866	8.300	8.555 ± 0.270
				8.300	7.966	8.266	8.933	8.033	8.100	8.266 ± 0.352
				8.433	8.400	8.233	8.766	8.600	8.100	8.422 ± 0.241
				8.300	8.366	8.166	8.633	8.666	7.700	8.305 ± 0.354
				8.266	8.433	8.200	8.566	8.533	8.066	8.344 ± 0.199
				7.866	7.133	8.000	7.566	7.466	7.766	7.633 ± 0.313
				8.600	8.600	8.766	9.233	9.066	8.300	8.761 ± 0.341
				8.320 ± 0.247	8.270 ± 0.440	8.263 ± 0.203	8.653 ± 0.605	8.506 ± 0.454	7.973 ± 0.274	
				0.81***	0.68**	0.37	0.76**	0.83***	0.44*	

Correlations between D (or E) -scores and mean values in each event are also indicated. *p < 0.05, **p < 0.001, ***p < 0.0001

All WCGs in this study have won medals in gymnastics world championships since 2015

*D-score* difficulty score, *E-score* execution score, *DTB* Deutscher Turner-Bund, *WGC* World Gymnastics Championships, *WC* World Cup, *IJGC* International Junior Gymnastics Competition, *SD* standard deviation

### MRI acquisition

Magnetic resonance imaging (MRI) data were obtained using a 3-T scanner (MAGNETOM Prisma, Siemens Healthcare, Erlangen, Germany) equipped with a 64-channel head coil. T1-weighted images (T1-WI) were acquired by a three-dimensional (3D) magnetization-prepared rapid gradient-echo sequence (MPRAGE). The acquisition parameters were: repetition time, 2300 ms; echo time, 2.32 ms; inversion time, 900 ms; voxel size, 0.90×0.90×0.90 mm^3^; and acquisition time, 5.31 min.

### GM volume measurement methods

#### VolBM

VolBM was performed using FreeSurfer version 5.3.0 (freesurfer-Linux-centos6_x86_64-stable-pub-v5.3.0; https://surfer.nmr.mgh.harvard.edu/fswiki), as previously described [14]. FreeSurfer was run with the recon-all pipeline using the default analysis settings on each T1-WI. Briefly, this pipeline included motion correction and intensity normalization of T1-WI, removal of non-brain tissue, automated Talairach transformation, segmentation of the subcortical WM and deep GM volumetric structures (including the hippocampus, amygdala, caudate, putamen, and ventricles), tessellation of the GM–WM boundary, and derivation of cortical thickness. After image processing, we extracted volumes for the cortical structures of the 34 bilateral Desikan–Killiany atlas [15] regions. Volumes of structures were then measured by multiplying the size of each voxel by the number of voxels.

### Statistical analysis

All statistical analyses were performed using IBM SPSS Statistics for Windows, Version 22.0 (IBM Corporation, Armonk, NY, USA). The Shapiro–Wilk test was used to assess the normality of the data, whereas the data on demographics were analyzed using an unpaired Student’s *t*-test and *χ*^2^ test for continuous and categorical variables, respectively. Statistical significance was set to 0.05.

During the VolBM analysis, the Mann–Whitney U test was used to compare the mean (bilateral hemispheres) normalized GM volumes (nGM; GM volume/intracranial volume) between the WCGs and controls. Then, the Benjamini–Hochberg false discovery rate (FDR) correction was applied to correct for multiple testing (34 regions of interest) with the level of significance for two-tailed *p* values set to 0.05. The relationship between the D-scores for individual gymnastic events (floor exercise, horizontal bar, rings, vault, parallel bars, and pommel horse) or the average D-scores from these individual scores and areas showing significant FDR-corrected p-values in group comparisons were evaluated using the Spearman’s rank correlation test, with the level of significance set to FDR-corrected *p*<0.05. The relationship between the D-scores from individual gymnastic events and average D-scores was also evaluated using Pearson correlation analysis at a significance level of *p*<0.05.

## Results

No significant difference was observed with regard to age (*p*=0.35) and sex (*p*=1.00) between the WCG and control groups. In the WCGs, the best D-scores for each gymnastic event and the average of the D-scores from a recent world gymnastics competition are shown in Table 1. Significant correlations between the average D-score and the D-scores from the rings event (*p*=0.0016, R^2^=0.73) or the parallel bars event (*p*=0.0029, R^2^=0.69) were found in the WCGs.

**Fig. 1:**
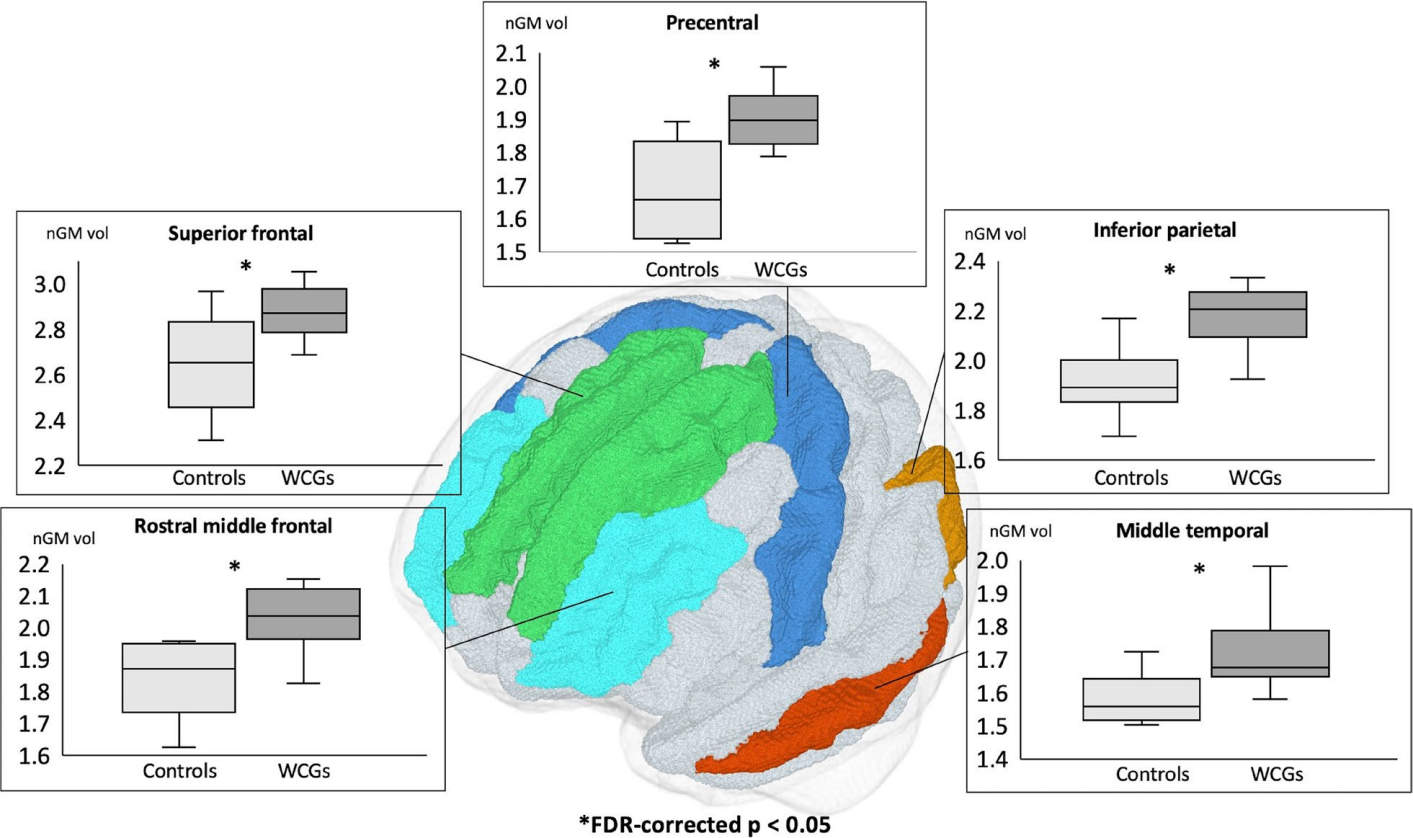
Comparison between normalized gray matter volumes of world-class gymnasts and healthy controls obtained using volume-based morphometry. The WCGs exhibit significantly (false discovery rate [FDR]-corrected *p* < 0.05) higher nGM volumes compared with the controls. *nGM* normalized gray matter, *HC* healthy controls, *WCG* world-class gymnasts

**Fig. 2:**
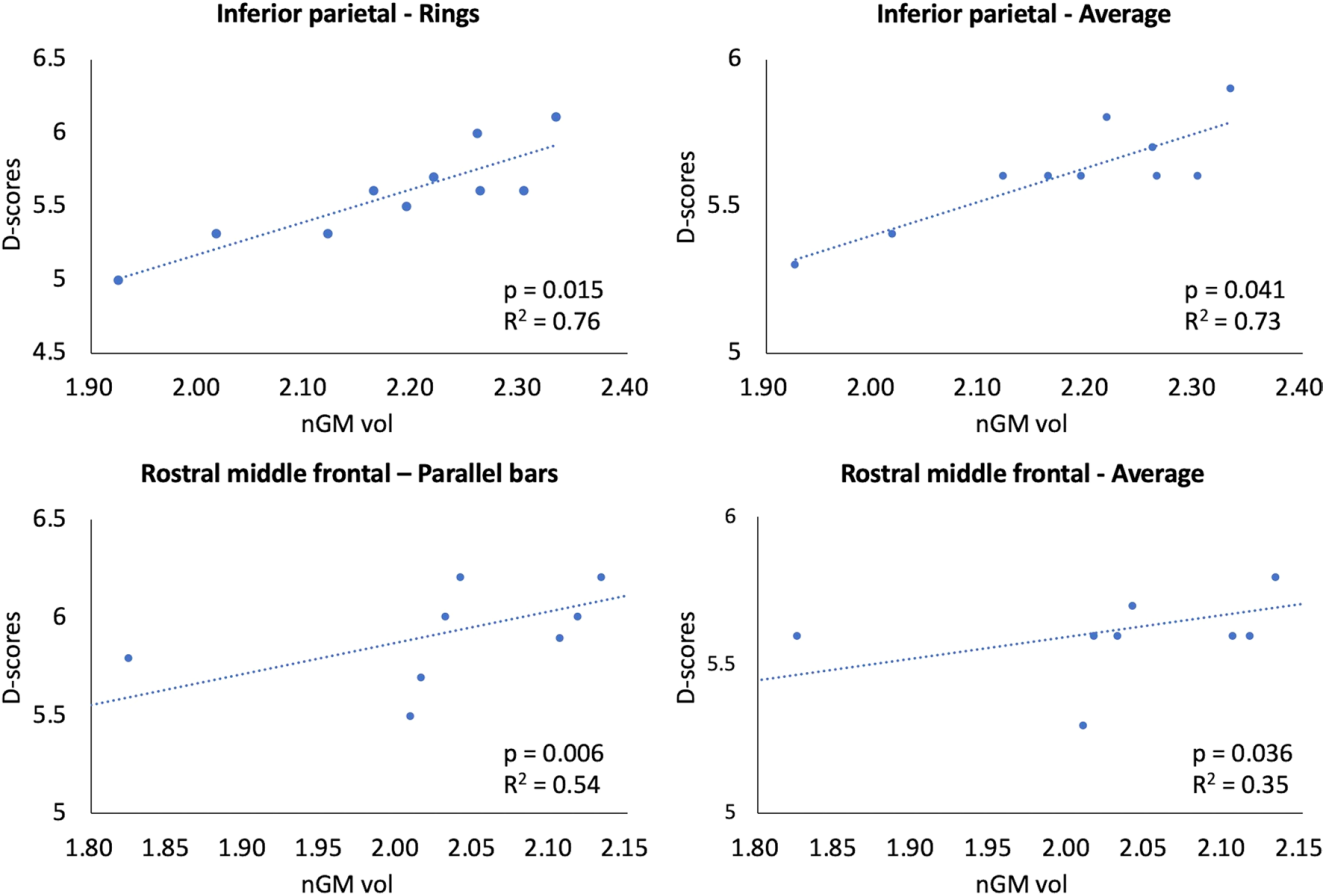
Correlation between normalized gray matter volumes obtained using volume-based morphometry in world-class gymnasts and the average or absolute D-scores for each gymnastics event. Statistically significant positive correlation between nGM volume in the inferior parietal and rostral middle frontal regions and the D-score from the rings event and average D-scores and D-score of parallel bars event and average D-scores, respectively. *nGM* normalized gray matter, *WCGs* world-class gymnasts

VolBM analysis showed statistically significant increases in mean nGM volumes (FDR-corrected *p*<0.05) in the inferior parietal lobule (IPL; *p*=0.036), middle temporal gyrus (MTG; *p*=0.044), precentral gyrus (PrG; *p*=0.044), rostral middle frontal gyrus (RMFG; *p*=0.044), and superior frontal gyrus (SFG; *p*=0.047) of WCGs compared with controls (Fig. 1, Table 2). Furthermore, the nGM volume of IPL was positively correlated with the D-score from the rings event (*p*=0.015, R^2^=0.76) and the average D-scores (*p*=0.041, R^2^=0.73), and that of the RMFG was also positively correlated with the D-score from the parallel bars event (*p*=0.006; R^2^=0.54) and average D-scores (*p*=0.036, R^2^=0.35) (Fig. 2).

**Table 2:** Normalized gray matter (nGM) volume of controls and world-class gymnast (WCGs) obtained using volume-based morphometry analysis

	Normalized gray matter volume				*p* value	FDR-corrected *p* value
	Controls		WCGs			
	Mean	SD	Mean	SD		
Bankssts	0.34	0.06	0.36	0.04	0.353	0.631
Caudal anterior cingulate	0.27	0.04	0.27	0.03	0.684	0.895
Caudal middle frontal	0.74	0.07	0.73	0.13	0.912	0.971
Cuneus	0.41	0.04	0.43	0.07	0.684	0.895
Entorhinal	0.22	0.04	0.22	0.02	0.796	0.933
Fusiform	1.50	0.14	1.50	0.10	0.529	0.817
Inferior parietal	1.92	0.15	2.18	0.13	0.001	0.036*
Inferior temporal	1.46	0.14	1.68	0.17	0.009	0.051
Isthmus cingulate	0.39	0.02	0.38	0.05	0.143	0.406
Lateral occipital	1.49	0.14	1.58	0.12	0.247	0.526
Lateral orbito-frontal	0.91	0.06	0.98	0.06	0.035	0.121
Lingual	0.91	0.11	1.00	0.12	0.190	0.498
Medial orbito-frontal	0.64	0.07	0.64	0.03	0.684	0.895
Middle temporal	1.55	0.15	1.72	0.12	0.005	0.044*
Parahippocampal	0.31	0.03	0.33	0.04	0.280	0.560
Paracentral	0.47	0.04	0.54	0.06	0.035	0.121
Pars opercularis	0.60	0.06	0.57	0.06	0.579	0.856
Pars orbitalis	0.28	0.02	0.32	0.03	0.011	0.056
Pars triangularis	0.54	0.06	0.54	0.07	0.971	0.971
Pericalcarine	0.28	0.05	0.29	0.05	0.481	0.779
Postcentral	1.24	0.21	1.35	0.09	0.063	0.195
Posterior cingulate	0.48	0.07	0.49	0.04	0.971	0.971
Precentral	1.42	0.14	1.46	0.08	0.003	0.044*
Precuneus	0.33	0.04	0.32	0.03	0.247	0.526
Rostral anterior cingulate	1.75	0.17	1.76	0.12	0.315	0.595
Rostral middle frontal	1.62	0.15	1.62	0.15	0.005	0.044*
Superior frontal	1.51	0.17	1.48	0.11	0.007	0.047*
Superior parietal	0.10	0.02	0.11	0.02	0.971	0.971
Superior temporal	0.28	0.04	0.33	0.04	0.796	0.933
Supramarginal	0.16	0.03	0.14	0.03	0.971	0.971
Frontal pole	0.89	0.05	0.89	0.06	0.393	0.668
Temporal pole	0.34	0.06	0.36	0.04	0.015	0.062
Transverse temporal	0.27	0.04	0.27	0.03	0.218	0.526
Insula	0.74	0.07	0.73	0.13	0.796	0.933

^*^Significantly higher nGM volume in WCGs compared to controls (false discovery rate [FDR]-corrected *p* < 0.05)

## Discussion

Using VolBM analysis, this study revealed that the nGM volumes of the IPL, MTG, PrG, RMFG, and SFG were higher in WCGs than in controls. Accumulating evidence has shown that increased GM volumes found in exercise-trained subjects are associated with increased functional plasticity and cognitive performance [2, 11, 16, 17]. Our findings support the concept that long-term motor skill training is associated with a complex bihemispheric cortical–subcortical network [2, 18]. The brain areas identified in this study are functionally related to motor control (i.e., PrG) [19, 20], interpretation of sensory information (i.e., IPL) [21–23], body image (i.e., IPL) [21, 22], spatial perception (i.e., IPL and MTG) [23, 24], spatial attention (i.e., IPL) [21–23], vision (i.e., IPL and MTG) [21–24], executive function (i.e., RMFG and IPL) [22, 25–27], and working memory (i.e., RMFG and SFG) [25–28]. A unique feature of gymnastics events is that each requires acrobatic performances comprising speed, strength, and flexibility; these physiological functions are all critical to supporting elaborate body movements. The increased GM volume in the IPL, MTG, PrG, RMFG, and SFG of WCGs is likely to result from many years of intensive training. These findings support a previous report, which suggested that, compared with non-gymnasts, Chinese gymnasts exhibit higher volumes of GM in somatosensory, motor control, and visuospatial areas [11]. The underlying mechanisms associated with training-dependent GM volume increases remain unknown, but they may involve microstructural changes in brain cells, including neurons, glial cells, and endothelial cells, induced by increased blood flow, oxygenation, and the production of brain neurotrophic factors and their receptors [2, 11, 16].

In WCGs, we analyzed correlations between the D-scores for each gymnastics event and the GM volumes of particular brain areas identified as containing more GM in gymnasts than controls. The D-scores for the rings and parallel bars events were found to be related to the GM volumes of the IPL and RMFG, respectively. The IPL is known to be involved in various neural functions, including spatial attention, multimodal sensory integration, body image, oculomotor control, and hand–eye coordination [21–23]. The RMFG is critical for executive function and working memory [25–27]. Working memory is a series of memory operations that involve manipulating and utilizing information while temporarily retaining it in the brain. Executive function is a cognitive system that controls thoughts and actions when performing complex tasks, and working memory is included in this process.

The rings represent the only apparatus that is continually in motion. Hence, this particular event requires prodigious body balance and muscle strength to halt body movements suddenly and allow the gymnast to maintain the position. Considering our findings, a high level of IPL function may be required to follow the precise movements of the rings, adjust body balance, and hold the body in an optimal position. On the parallel bars, techniques involve hand-release from and regrasping of the bars. Therefore, a high level of visuospatial working memory may be required to identify the precise location of the bar/bars to be regrasped quickly, and this level of function may be associated closely with the level of competitive ability seen in WCGs. Structural changes in the IPL and RMFG are also likely to be the result of intensive training in WCGs.

Because the average D-score reflects the level of skill demonstrated in the individual all-around event, correlations were determined between the average D-score and GM volumes of particular brain areas identified as containing more GM in gymnasts than in controls. Interestingly, significant positive correlations were found only in the IPL and RMFG, indicating that the brain areas and functions associated with the D-scores from the rings and parallel bars were also involved in determining the average D-score. Indeed, the average D-scores were highly correlated with the D-scores for both the rings and parallel bars but not the other events. These characteristics of the WCGs who participated in this study may have caused the IPL and RMFG to be identified as brain areas that affect the average D-score. However, although each gymnastic event entails specific skills, all events involve common body movements, including handstands, somersaults, and twist techniques, all of which require complex motor skills, based on sensory perception–motor integration, to adjust balance and hold the body optimally. Therefore, our finding that the IPL and RMFG affect the average D-score may suggest that higher levels of spatial attention, multimodal sensory integration, body image, oculomotor control, hand–eye coordination, executive function, and working memory facilitate sensory perception–motor integration and elaborate body movements required to undertake all gymnastics events, thus enabling the competitor to obtain a higher score in the individual all-around event.

## Limitations

Since the number of Japanese WCGs who are currently active is extremely limited, the sample sizes in this study were small (10 WCGs and 10 controls). However, to confirm whether genetic factors affect the GM volumes of particular brain areas in WCGs and/or whether long-term intensive training is a main factor that contributes to the structural changes, future investigations using longitudinal studies with a large number of WCGs and/or control subjects will be required.

## Conclusions

Overall, we found that the GM volumes of particular brain areas play an indispensable role in gymnastic ability and are closely associated with individual D-scores for specific gymnastic events in WCGs. The increased GM volumes in these areas may represent the neural basis for outstanding gymnastics performance resulting from brain plasticity. GM volumes in these brain areas of interest could become markers for the objective evaluation of gymnastic performance. In this regard, our findings demonstrate that GM volumes in the IPL and RMFG may be potential markers for evaluating the performance of a gymnast on the rings, parallel bars, or in the individual all-around event.

## Data Availability

The datasets used and/or analyzed during the current study are available from the corresponding author on reasonable request.

## Abbreviations

3D: Three-dimensional
D-score: Difficulty score
FDR: False discovery rate
FIG: International Gymnastics Federation
GM: Gray matter
MPRAGE: Magnetization-prepared rapid gradient-echo sequence
MRI: Magnetic resonance imaging
nGM: Normalized GM
T1-WI: T1-weighted images
VolBM: Volume-based morphometry
WCGs: World-class gymnasts
WM: White matter
IPL: Inferior parietal lobule
MTG: Middle temporal gyrus
PrG: Precentral gyrus
RMFG: Rostral middle frontal gyrus
SFG: Superior frontal gyrus
VBM: Voxel-based morphometry
